# Sex Differences in Hierarchical and Modular Organization of Functional Brain Networks: Insights from Hierarchical Entropy and Modularity Analysis

**DOI:** 10.3390/e26100864

**Published:** 2024-10-14

**Authors:** Wenyu Chen, Ling Zhan, Tao Jia

**Affiliations:** College of Computer and Information Science, Southwest University, Chongqing 400715, China; c3323867@email.swu.edu.cn (W.C.); zl0327@email.swu.edu.cn (L.Z.)

**Keywords:** sex difference, hierarchical organization, modular organization, hierarchical entropy, functional brain network

## Abstract

Existing studies have demonstrated significant sex differences in the neural mechanisms of daily life and neuropsychiatric disorders. The hierarchical organization of the functional brain network is a critical feature for assessing these neural mechanisms. But the sex differences in hierarchical organization have not been fully investigated. Here, we explore whether the hierarchical structure of the brain network differs between females and males using resting-state fMRI data. We measure the hierarchical entropy and the maximum modularity of each individual, and identify a significant negative correlation between the complexity of hierarchy and modularity in brain networks. At the mean level, females show higher modularity, whereas males exhibit a more complex hierarchy. At the consensus level, we use a co-classification matrix to perform a detailed investigation of the differences in the hierarchical organization between sexes and observe that the female group and the male group exhibit different interaction patterns of brain regions in the dorsal attention network (DAN) and visual network (VIN). Our findings suggest that the brains of females and males employ different network topologies to carry out brain functions. In addition, the negative correlation between hierarchy and modularity implies a need to balance the complexity in the hierarchical organization of the brain network, which sheds light on future studies of brain functions.

## 1. Introduction

Biological sex is a crucial variable in the field of neuroscience because it relates both the neural mechanisms underlying daily life and the incidence of neuropsychiatric disorders [1,2,3]. For instance, females generally exhibit more activation in the prefrontal regions implicated in affective processing, which may result in better performance in terms of emotion regulation [4,5] and higher susceptibility to emotional disorders such as anxiety and depression [6,7,8,9,10]. In contrast, males typically display higher synchronization in brain regions such as the bilateral posterior cingulate and left middle occipital gyrus, which contribute to better performance in mental rotation tasks [11], but may give rise to higher risk for autism spectrum disorder (ASD) [12] and attention deficit hyperactivity disorder (ADHD) [13]. A understanding on a neural basis of the sex differences in healthy adults may provide further explanations of the neural mechanisms of daily life activities and neuropsychiatric disorders.

Recently, advanced neuroimaging techniques like functional magnetic resonance imaging (fMRI) [14] and theories like network science have emerged as powerful tools for elucidating the sex differences in the topological properties of functional brains network [15,16]. Many studies on sex differences have started to look into the functional connectivity of the brain network and have found substantial differences in connection strength [17,18,19]. While using connection strength is straightforward, it only provides a very simple perspective on the brain network. To further explore the functional brain network, topological properties, such as the hierarchical structure and modular structure of the network, have become points of interest [20]. Several studies report stronger intra-module connections in females and stronger inter-module connections in males [17,19], implying that males and females may have different hierarchical organization in their brain networks.

Modules are defined as highly connected clusters of entities that are sparsely connected to outside entities. They are believed to come from the hierarchy of the network because a pure random network does not exhibit modular structures [21]. Hierarchy and modularity offer new perspectives for exploring brain functions across various scales [22,23,24]. For example, research utilizing features reveals specific age-related effects on the segregation of modules within brain networks [25,26]. Several studies have highlighted that this type of hierarchy significantly enhances the brain’s ability to process complex information, and greatly improves efficiency, robustness, and adaptability [22,27,28]. However, current research primarily focuses on exploring modularity or hierarchy [23,29], with little attention given to their inter-relationships. Investigating these relationships could provide us with a more comprehensive understanding of brain network topology across various scales. Moreover, studies on sex differences predominantly concentrate on modularity [17,30,31], lacking detailed analyses of the differences in hierarchy between female and male brain networks.

In addition, current studies on sex differences usually focus on the positively correlated brain network, with only a few exceptions considering the negatively correlated brain network [32]. But recent findings have highlighted the pathophysiological significance of negative connections in the resting state, particularly between the default mode network and the dorsal attention network [33,34,35,36]. Positive connections during rest may signify functional integration, whereas negative connections could denote functional segregation [37,38]. Positive and negative connections probably play equally important roles in brain function. Therefore, functional brain networks with both positive and negative strength should be considered for a more thorough investigation of sex differences.

In this study, we investigate the correlations between hierarchy and modularity in brain networks that include positive and negative connections, and elucidate sex-based differences in hierarchical and modular structures. Specifically, we employ hierarchical clustering to generate dendrograms for each individual. We calculate the hierarchical entropy, which reflects the complexity of hierarchical structures, and the maximum modularity, which is associated with the connection strength within the optimal partition. We identify a negative correlation between the hierarchical entropy and the maximum modularity. We find a significant difference between males and females. On average, male brain networks demonstrate a more complex hierarchy, whereas female brain networks have stronger connectivity within the module. Moreover, we generate co-classification matrices for each group to analyze, in detail, the difference in hierarchy between sexes [39]. Females’ and males’ brain networks display different interaction patterns between VIN and DAN. Our findings provide new evidence on sex differences in the brain network, providing a foundation for future studies of brain function, cognitivity, and disorders.

## 2. Materials and Methods

### 2.1. Subjects and Acquisition

We utilized resting-state fMRI data from the Southwest University Longitudinal Imaging Multimodal (SLIM) project, which has been approved by the Institutional Review Board of the Brain Imaging Center at Southwest University, Chongqing, China. The resting-state functional MRI images were collected using a Siemens Trio 3.0T scanner (Siemens Medical, Erlangen, Germany) at the Southwest University China Center for Brain Imaging. They were acquired using a single-shot, gradient-recalled echo planar imaging sequence (repetition time =2000 ms, echo time =30 ms, flip angle $=90^{\circ }$, field of view (FOV) =220×220 mm, thickness/slice gap =3/1 mm, and voxel size =3.4×3.4×3 mm^3^). All subjects were instructed to rest with closed eyes and not think of anything in particular. For a detailed description of the subject information and data acquisition parameters, please see [40]. Subjects were excluded if they (a) were <18 years of age; (b) had <200 timepoints of acquisition time; (c) lacked sex information; (d) had taken psychiatric drugs for psychiatric or neurological disorders; or (e) had a history of head trauma. The final dataset comprised N=541 subjects (299 females, mean age: 19.76±0.03; 241 males, mean age: 19.75±0.03; range: 18–27 years).

### 2.2. Construction of the Functional Brain Network

**Figure 1:**
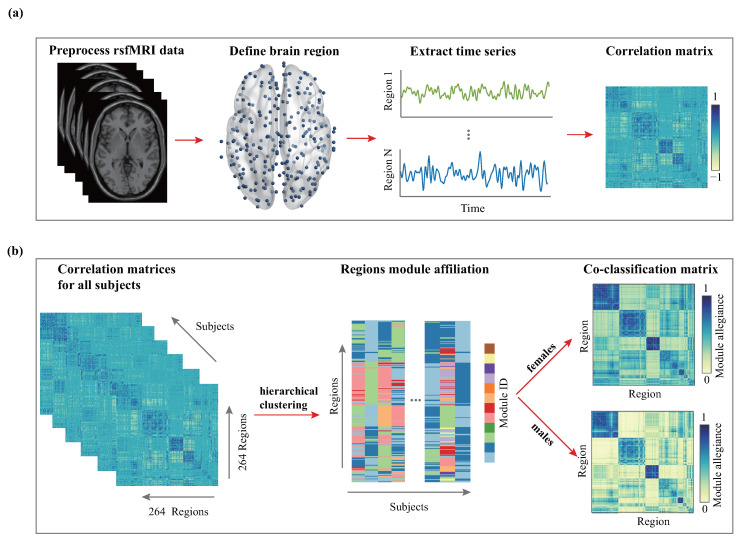
Procedure for constructing a functional brain network and co-classification matrix. (**a**) Construction of the functional brain network. Standard preprocessing steps, such as motion correction, normalization, and spatial smoothing, were applied to the resting-state fMRI data to ensure high-quality input for subsequent analyses. The cerebral cortex was divided into 264 brain regions based on the power template [41]. Each region served as a node in the brain network. The mean time series of each region was extracted. Pearson’s correlation coefficients were calculated for each pair of nodes, resulting in a 264×264 correlation matrix that represents the functional brain network. (**b**) Construction of the co-classification matrix. In the first stage, the modular structure for each individual’s brain network was detected using hierarchical clustering and maximum modularity. In the second stage, a co-classification matrix for each group (i.e., females and males) was constructed by combining all the individual modular structures.

An overview of the processing of the construction of the functional brain network for each individual is displayed in Figure 1a, with each step explained below.

#### 2.2.1. Preprocessing rsfMRI Data

Functional images were preprocessed using the DPARSF [42] and SPM8 toolkits [43]. After discarding the first 10 rs-fMRI images to ensure signal stabilization, the rest of the rs-fMRI images were corrected for slice time difference and head motion. Next, we registered the images and spatially normalized them to the Montreal Neurological Institute (MNI) template, and resampled the voxel size to $=3\times 3\times 3\;{\mathrm{mm}}^{3}$. We then performed spatial smoothing with a 6mm full-width at half maximum Gaussian kernel (FWHM =6mm). We cleared confounding signals, including white matter, cerebrospinal fluid signals, and head motion effects, using the Friston 24-parameter model [44,45]. Finally, we applied a temporal band-pass filter (0.01–0.1 Hz) to reduce high-frequency physiological noise.

#### 2.2.2. Constructing Network

Nodes in functional brain networks are defined by the Power atlas [41], which includes 264 brain regions. This atlas also offers a parcellation scheme for these nodes, delineating 13 functional modules corresponding to known large-scale brain networks coherent during both task activity and rest. The 13 functional modules include the auditory network (AUN), visual network (VIN), subcortical network (SUB), salience network (SN), cerebellar (CER) network, default mode network (DMN), ventral attention network (VAN), dorsal attention network (DAN), cingulo-opercular task control (COTC), fronto-parietal task control (FPTC), sensory/somatomotor hand network (SMH), and sensory/somatomotor mouth network (SMM). For each subject, the brain network is quantified by a 264×264 matrix A, where each element $a_{i,j}$ is calculated using the Pearson correlation of the averaged Blood Oxygenation Level-Dependent (BOLD) signals (i.e., averaged time series) between node *i* and node *j*. To eliminate the possible noise from insignificant connections, we apply the proportional threshold method [46]. First, we use the false discovery rate (FDR) correction to mark statistically insignificant connections (p>0.001). Then, we count, in each sex, the ratio in which a link is insignificant. If over 50% of the time, a link between nodes *i* and *j* is marked as insignificant, we set $a_{i,j}=0$ for all individuals within the same sex. A threshold value of 50% was chosen according to [46,47].

### 2.3. Hierarchical Clustering

Hierarchical clustering is a basic and extensively utilized approach for delineating the multiscale modular hierarchy in brain networks [48,49,50,51]. This technique facilitates the exploration of how entities are organized into modules, thereby illuminating the complexity of hierarchical structures within these networks [52]. In this study, we adopt a hierarchical agglomerative clustering method, which is particularly effective in capturing the interactions among brain regions. Given the fact that both positive and negative weights exist in the brain network connections, we first transform the connection matrix A into a distance matrix D by assigning the element value $d_{i,j}=1-a_{i,j}$. Then, we perform the standard hierarchical clustering on D to produce a dendrogram. At each iteration, two entities (nodes or clusters) with the smallest distance are merged together to form a new cluster, adding a new layer to the dendrogram. The distance between the two entities is calculated as the average distance of the nodes in them. This process is repeated until all nodes are merged into a single cluster.

### 2.4. Network Topology Analysis

The complexity and connectivity within brain networks can be explored through various metrics, each highlighting different aspects of network dynamics [53]. In the following, we briefly introduce the metrics applied in this study. We also introduce the tool used to obtain the aggregated module in order to obtain detailed information on the brain region differences between females and males.

#### 2.4.1. Hierarchical Entropy

Entropy is widely used as a measure of randomness. To quantify the complexity of a hierarchical organization, several metrics are proposed, such as thermodynamic entropy, Kolmogorov entropy, and hierarchical entropy [54,55,56,57,58,59].

Hierarchical entropy [55,56] is a metric that focuses on the process of hierarchical clustering. For the *i*th layer of the dendrogram, we can quantify the uniformity of the cluster size using Shannon-entropy as follows:

(1) $$h_{i}=-\sum_{j\in J}P_{ij}\log_{2}P_{ij},$$

where *J* is the set of clusters on the *i*th layer, $n_{ij}$ is the number of nodes of cluster *j* on the *i*th layer, $P_{ij}=\frac{n_{ij}}{N}$ is the size distribution of the cluster, and *N* is the total number of nodes. $h_{i}$ is high if multiple small clusters with similar sizes are formed, and low if a few big clusters dominate. Hence, the value of $h_{i}$ reflects the formation of the hierarchical structure. By averaging the $h_{i}$ of each layer, we have the hierarchical entropy *H* of a hierarchical organization:

(2) $$H=\sum_{i}^{N-1}\frac{h_{i}}{N-1}.$$

#### 2.4.2. Modularity

Modularity is a metric used to assess the extent to which modules or communities are separated [60,61]. Higher modularity suggests that the modules are further apart such that the connections within each module are stronger than those in between. The maximum modularity of a network usually refers to the best partition of nodes. In this work, we consider the maximum modularity $Q^{*}$ as a measure of the network partition:

(3) $$Q^{*}=max\left\{\frac{1}{2W^{+}}\sum_{ij}\left(w_{ij}^{+}-e_{ij}^{+}\right)\delta \left(m_{i},m_{j}\right)-\frac{1}{2W^{+}+2W^{-}}\sum_{ij}\left(w_{ij}^{-}-e_{ij}^{-}\right)\delta \left(m_{i},m_{j}\right)\right\},$$

where *W* is the total weight of all connections, $w_{ij}$ is the weighted and signed connection between region *i* and *j*, $e_{ij}$ is the strength of a connection divided by the total weight of the network, and $\delta (m_{i},m_{j})$ is set to 1 when regions *i* and *j* are in the same module, and 0 otherwise. “+” and “−” superscripts denote all positive and negative connections, respectively.

### 2.5. Linear Regression Analysis

We performed a linear regression analysis to investigate the relationship between the modularity ($Q^{*}$) and the hierarchical entropy (*H*), using $Q^{*}$ as the independent variable and *H* as the dependent variable. The significance of the regression coefficient was tested using a *t*-test, with a significance threshold of p<0.05. This analysis was implemented using the SciPy library in Python.

### 2.6. Co-Classiffication Matrix

The co-classification matrix C, also known as the module allegiance matrix [39], is a tool used to merge different partition structures and reach a consensus modular structure [62,63]. More specifically, we found the module structure of the brain network for each individual by performing hierarchical clustering and cutting the dendrogram at the maximum modularity. For the groups of females and males, we calculated $c_{ij}$ which is the probability that nodes *i* and *j* were assigned to the same functional module over all subjects, which eventually yielded the co-classification matrix C (Figure 1b). The element values of the matrix effectively reveal the inherent overlaps in the brain network architecture across individuals of the same sex. Nodes that often appear in the same module will have a high connection strength in C, whereas nodes that are rarely in one module will have a low connection strength. Therefore, when performing hierarchical clustering on C, the modular structure obtained reflects a consensus partition that is “averaged” over multiple individuals [64,65,66]. As an example, if nodes 1, 2, and 3 are consistently assigned to the same module, we will have $c_{12}=c_{21}=c_{13}=c_{31}=c_{23}=c_{32}=1$, and the other elements will equal 0. The hierarchical clustering of C will yield a module that contains nodes 1, 2, and 3, which perfectly reflects the modular structure in the population.

### 2.7. Statistical Tests

A two-sample *t*-test (two-tailed) was used for mean comparisons between the female and male groups, with the significance thresholds set to p<0.05. To calculate the effect size of Cohen’s *d* value for each *t*-test, we doubled the t-value and then divided it by the square root of the degrees of freedom. A positive *d* value indicates a stronger effect in females, while a negative *d* value indicates a stronger effect in males. The larger the *d* value, the more significant the effect.

## 3. Results

### 3.1. The Relationship between *H* and $Q^{*}$

Several works analyze the association between the hierarchical structure and modularity of the brain network. Some studies report a positive correlation between them [23,24]. But other studies suggest that modularity may be reduced due to dense inter-layer connections, potentially giving rise to a negative correlation [27]. To re-evaluate this relationship, we employed linear regression to quantify the relationship between *H* and $Q^{*}$.

**Figure 2:**
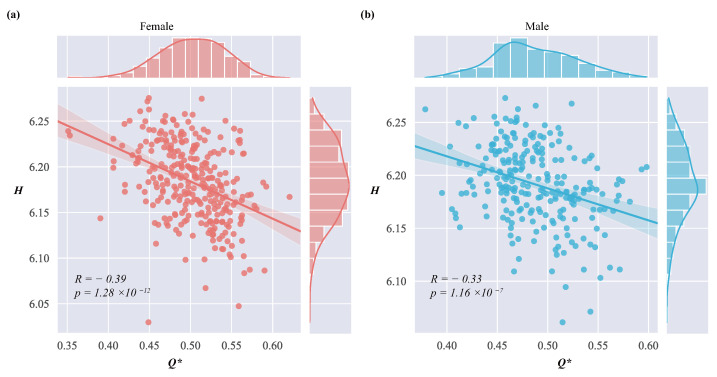
Relationship and regression results between *H* and $Q^{*}$. The correlation coefficient *R* was calculated between *H* and $Q^{*}$, and linear regression was performed, represented by a solid line. (**a**) Females. (**b**) Males.

Figure 2 depicts a significant negative correlation between *H* and $Q^{*}$ in brain networks. When the complexity of the hierarchical structure becomes high, the modular structure becomes diminished. On the contrary, when the hierarchical structure becomes simpler, the modular structure is more distinct. This inverse relationship suggests a competitive dynamic between hierarchical complexity and modular clarity, potentially impacting both their functional efficiency and robustness.

### 3.2. A Comparison between the Sexes at the Mean-Level

**Figure 3:**
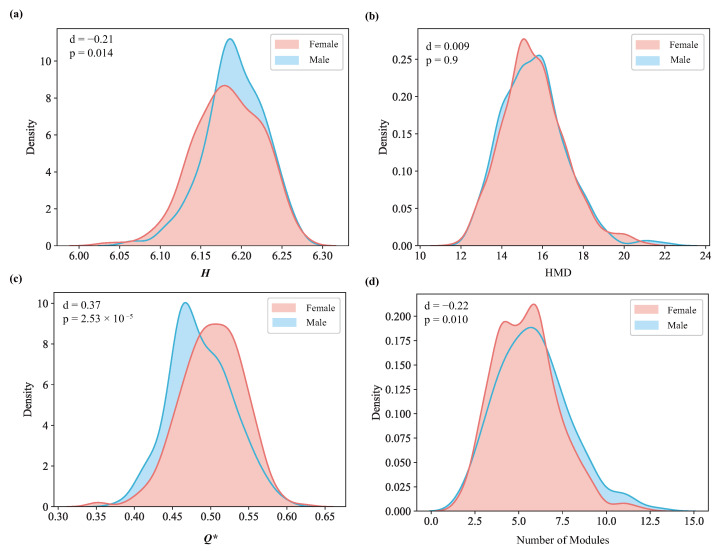
Comparison of the distributions of *H*, HMD, $Q^{*}$, and Number of Modules between females and males. For all figures, *d* is the effect size and *p* is the *p*-value of the two-sample *t*-test. (**a**) The kernel density of *H* for each sex. (**b**) The kernel density of the maximum depth of hierarchical organization for each sex. (**c**) The kernel density of $Q^{*}$ for each sex. (**d**) The kernel density of the number of modules.

We first compared the mean of *H* between sexes and identified a significant difference (Figure 3a). The brain network of males exhibits more complex hierarchical structures than that of females. To further investigate the reasons for this difference, we computed the hierarchical maximum depth (HMD) [67], defined as the maximum distance from the root node to a leaf node in the dendrogram. If the dendrogram is formed such that each node iteratively merges to a single core cluster, the HMD will be very high. On the contrary, if small clusters pair with each other at each layer, the HMD will be low. We found no significant difference in HMD between sexes (Figure 3b). Therefore, the different complexities in the hierarchical structures do not originate from different heights of the dendrogram. The uniformity of cluster sizes at each layer, related to how the modules are formed, plays a more important role.

To further understand the differences in hierarchical structures, we identified the maximum modularity $Q^{*}$ and cut the dendrogram according to $Q^{*}$ to obtain the modules of the network. We found that females display a significantly higher $Q^{*}$ (Figure 3c), indicating stronger intra-modular connectivity than males. Males display a greater number of modules (Figure 3d), suggesting broader, more dispersed network organization.

Overall, these findings imply that males have more dispersed network organization, while females have a more integrated and coordinated network.

### 3.3. A Comparison between the Sexes at the Consensus Level

**Figure 4:**
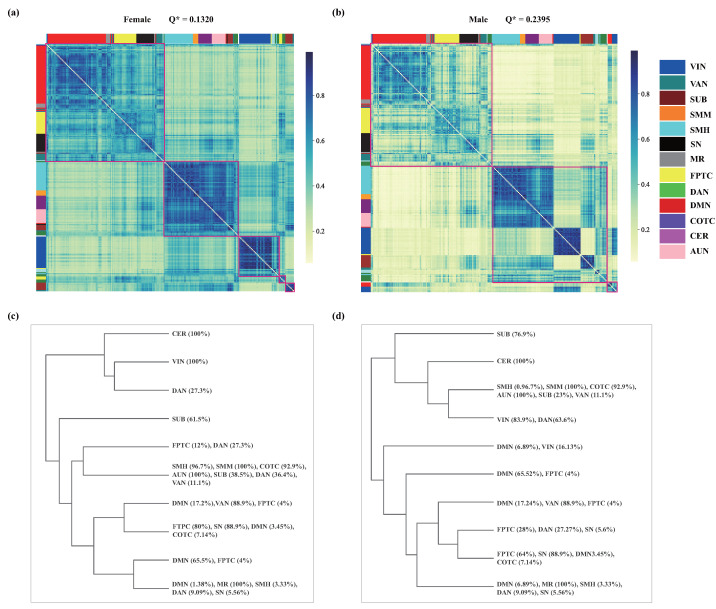
The co-classification matrix and its hierarchical organization. (**a**,**b**) The co-classification matrix for each sex. Red boxes represent the modular partition. The color reflects the value of the matrix element, which suggests that the modular structure is more stable in females than in males. (**c**,**d**) We extracted the module compositions from the co-classification matrix for females and males, and the figure shows the top 10 modules. The percentages represent the proportion of nodes from each functional network within these modules. For example, VIN (100%) signifies that all brain regions associated with VIN are included in the same module.

To assess the details of the modular difference between females and males, we constructed a co-classification matrix and applied hierarchical clustering to obtain the consensus partition for each sex (Figure 4a,b). Overall, the element value of the co-classification matrix is higher for females than males. This suggests that the modular structure in females is more stable than that in males. For the consensus hierarchical structure, females exhibit high inter-module co-occurrence, blurring the boundaries between modules and resulting in a low modularity value ($Q^{*}=0.1320$). But this high co-occurrence probability is also pronounced within smaller modules, eventually giving rise to five modules at the optimal partition. In contrast, males show high intra-module co-occurrence and low inter-module co-occurrence, which results in a higher modularity value ($Q^{*}=0.2395$) than that in females. Due to the comparable co-occurrence probabilities between smaller modules like SUB and VIN, and larger ones formed by SMH, SMM, COTC, and AUN, they are grouped into one large module. We identify three modules for males.

We further looked into the dendrogram and analyzed the formation of the modules in each sex (Figure 4c,d). We identify distinct interactions within the DAN and VIN brain regions. In females, the DAN region exhibits frequent collaboration both within and between modules, whereas the VIN regions engage more with other areas within the same VIN module. In males, a smaller subset of the DAN regions collaborates with specific parts of the FPTC regions, while a more substantial portion integrates extensively with the VIN regions. These observation suggest that females and males use different integration strategies to form hierarchical organization. In addition, we also observe some differences in the modular arrangements between the sexes. For instance, in females, modules including DMN, FPTC, SN, MR, and VAN show a higher co-occurrence probability compared to males, and areas within the DMN and VIN typically stay within their respective modules. In contrast, in males, a few nodes from DMN and VIN form an independent module. The SUB regions in females tend to interact internally, while in males, they are more likely to interact with other functional modules. Overall, the modular structure in females is more stable, exhibiting higher co-occurrence probabilities and less variability than in males.

## 4. Discussion

Utilizing the resting-state fMRI dataset, we investigate whether there are sex differences in the hierarchical organization and modular structure of brain networks, and further look into their specific differences. We employ hierarchical entropy (*H*) to quantify the complexity of the brain’s hierarchical organization and maximum modularity $Q^{*}$ to assess the network’s modularity. Our analysis indicates a negative correlation between *H* and $Q^{*}$. Specifically, female brain networks demonstrate higher $Q^{*}$, suggesting stronger intra-module connections, whereas male brain networks exhibit greater *H*, indicating more complex interactions among different clusters. Further, following a detailed examination of the co-classification matrix, we find higher interaction probabilities within female functional modules and more frequent between-functional-module interactions in males, particularly in DAN and VIN. These differences result in inconsistent modular structures between males and females, suggesting that these two sexes employ different organizational and integration strategies.

Our study quantitatively confirms an inverse correlation between the complexity of hierarchy and modularity in brain networks, aligning with prior findings [27]. More complex hierarchical structures enhance cross-regional communication and information integration [24,68], yet they might reduce the independence and local processing efficiency of modules. Conversely, while highly modular networks excel at processing local information and ensuring functional autonomy [23], they could impede long-distance information transfer and collaboration across modules. These insights emphasize the need for a balance between hierarchical complexity and modularity to optimize brain network functionality [22,69]. This balance enhances robustness and adaptability, which are crucial for responding to environmental changes and boosting cognitive performance, illustrating the critical interplay between network structure and brain function [27,28,37,46,70].

At the mean level, we observe that females exhibit lower hierarchical complexity and higher modularity, in contrast to males, who show higher hierarchical complexity and lower modularity. These observations are consistent with the negative correlation between hierarchical complexity and modularity, suggesting differing organizational strategies between sexes. This trend is further supported by the use of hierarchical clustering methods on the co-classification matrix, where we find that the female group tends to integrate more within functional modules, while the male group engages more in interactions between modules. Such patterns lead to simpler interaction patterns in females and more complex ones in males, explaining the observed differences in complexity. Additionally, the analysis of the consensus modular structure shows higher intra-modular co-occurrence in the female group compared to the male group, which may account for the greater modularity observed at the mean level. These findings are corroborated by previous studies that indicate stronger intra-modular connections in females and stronger inter-modular connections in males [17,19,71,72], supporting our conclusions on the distinct modular and hierarchical structures across sexes. These insights collectively underscore the profound impact of sex on brain network organization, reflecting distinct information processing strategies that have broad implications for understanding cognitive and behavioral sex differences [73,74,75].

Specifically, within the consensus hierarchical structure, we observe notable differences in integration styles within the VIN between the male group and the female group. In the female group, the VIN regions primarily engage in integration within the same functional module, indicating that this robust intra-modular integration underlies females’ enhanced abilities in processing visual details, recognizing facial expressions, and perceiving emotions [4,5,76]. On the contrary, the male group shows a tendency for the VIN regions to integrate more extensively with the DAN and the DMN. This pattern highlights males’ strengths in tasks that require spatial orientation and environmental awareness [11,77]. Such inter-modular integration is crucial for combining complex environmental information, which supports spatial navigation, multitasking, and rapid responses in urgent situations [78,79,80].

However, the current study also has several limitations. First, we apply the proportional threshold method for constructing the brain networks. Although the 50% threshold is the most commonly used, as it balances retaining network sparsity and reducing connections with lower consistency across individuals in the same group, recent studies have shown that the detection of group differences at the network level is highly dependent on the chosen threshold [46,47,81]. Future research will further explore the influence of sex on the topological characteristics of brain networks across a broader range of threshold levels. Second, although we observe most sex differences based on resting-state fMRI, it does not capture task-specific brain activation [82,83], which might be crucial for understanding the functional differences between male and female brains. Third, *H* provides a quantitative measure of complexity but does not offer insights into the functional implications of the observed differences. Higher or lower entropy values might not directly correlate with specific cognitive or behavioral traits, making it difficult to draw definitive conclusions about sex differences. Finally, although our findings reveal some significant sex differences in functional brain networks, we did not account for other factors, such as school years, psychology, cultural backgrounds, and socioeconomic factors, which may also influence the sex differences in brain function [84]. Given these limitations, future work should aim to combine analyses of the multi-scale hierarchical structures of resting-state functional brain networks with task-specific paradigms and sociocultural factors. This integrative approach may facilitate the establishment of a deeper understanding of sex differences in daily behaviors and cognitive functions.

## 5. Conclusions

In summary, this study conducts a systematic analysis of the relationship between the hierarchical organization and modular structure of brain networks, clearly illustrating the impact of sex on these structural attributes. Our findings identify a pronounced negative correlation: the more complex a brain network’s hierarchical organization, the lower its modularity. Notably, female brains demonstrate enhanced modularity, while male brains display more complex hierarchical structures. These distinctions underscore the significance of including sex considerations in neuroscience research, particularly when investigating the mechanisms by which the brain processes and integrates information. At the consensus level, we have observed that male and female brains employ distinct strategies for organization and integration, which highlights specific sex differences in how information is processed and integrated. Future research should delve deeper into how these sex-specific differences in brain network structures influence cognitive and behavioral functions. Furthermore, as these functional architectures may influence disease susceptibility and treatment responses, further research is essential to develop optimized prevention and intervention strategies based on sex differences.

## Data Availability

Data are contained within the article.
